# A Cross-Sectional Study on the Prevalence of Subclinical Hypothyroidism in Metabolic Syndrome Patients at a Tertiary Care Hospital

**DOI:** 10.7759/cureus.67851

**Published:** 2024-08-26

**Authors:** Gaurav Lakhani, Parth Patel, Tinkal C Patel

**Affiliations:** 1 Department of Cardiology, Institute of Post-Graduate Medical Education and Research and Seth Sukhlal Karnani Memorial Hospital, Kolkata, IND; 2 Internal Medicine, Chimanlal Ujamshibhai Shah Medical College, Surendranagar, IND; 3 Department of General Medicine, Dr. M. K. Shah Medical College and Research Center, Ahmedabad, IND

**Keywords:** thyroid dysfunction, subclinical hypothyroidism, metabolic syndrome, hypothyroidism, cardiovascular diseases, atherosclerosis

## Abstract

Background and objective: Metabolic syndrome (MetS) is defined as a “constellation” of cardiometabolic risk factors, characterized by hypertension, atherogenic dyslipidemia, hyperglycemia, prothrombotic and proinflammatory conditions which, jointly, increase the risk of suffering cardiovascular diseases (CVD) and type 2 diabetes mellitus. Thyroid dysfunction is also believed to affect parameters such as high-density lipoprotein (HDL) cholesterol, triglycerides, plasma glucose, and blood pressure, in turn increasing the risk of CVD. Subclinical hypothyroidism (SCH), which independently raises the risk of CVDs and their associated complications, is more frequently detected in patients with MetS compared to the general population. When both conditions coexist, the risk of CVD and its complications is significantly heightened. The objective of the study was to find out the prevalence of SCH in patients with MetS.

Methods: A prospective cross-sectional study was conducted in the Department of General Medicine, New Civil Hospital, Surat, Gujarat, India. Eighty patients who fulfilled the criteria for MetS by the National Cholesterol Education Program Adult Treatment Panel III (NCEP ATP III), were taken into the study. A detailed history, anthropometric measurements, blood pressure, fasting blood glucose, lipid profile, and thyroid profile (Free T3, Free T4, and serum thyroid-stimulating hormone (TSH)) were undertaken. The thyroid profile was done by chemiluminescence immunoassay (CLIA) method.

Results: Out of 80 patients with MetS, 48 were female and 32 were male, with the overall mean age of the study population being 46.5±9.5 years. Among them, 23.7% of the population was found to be having thyroid dysfunction. Among the thyroid dysfunction, SCH was highly prevalent (18.8%), 3.8% patients had overt hypothyroidism and 1.3% patients had subclinical hyperthyroidism. There were no overt hyperthyroid patients in our study. HDL (mg/dl) and TSH (mIU/L) were significantly higher in the SCH group as compared to other types of hypothyroidism group (p-value < 0.05).

Discussion: There is a statistically significant prevalence of SCH (18.8%) in MetS patients. It is clear from the study that one-fifth of MetS patients or every fifth patient with MetS had SCH. Thus, looking proactively for SCH in MetS and treating it would prevent conversion to overt hypothyroidism and complications of MetS.

## Introduction

Metabolic Syndrome (MetS) is defined as a “constellation” of cardiometabolic risk factors, characterized by hypertension, atherogenic dyslipidemia, hyperglycemia, prothrombotic and proinflammatory conditions which, together, increase the risk of suffering cardiovascular diseases (CVDs) and type 2 diabetes mellitus [1,2]. Thyroid dysfunction, prominently subclinical hypothyroidism (SCH) has been observed more frequently in MetS patients than general population [3]. Both MetS and hypothyroidism are independent risk factors for CVDs. The presence of both conditions may be compounded to increase the risk for CVD and a considerable overlap occurs in the pathogenic mechanisms of atherosclerotic CVD by MetS and hypothyroidism [4]. The prevalence of MetS (based on the National Cholesterol Education Program Adult Treatment Panel III (NCEP ATP III) criteria, 2001) varied from 8% to 43% in men and from 7% to 56% in women around the world [5]. The prevalence of MetS in India has been documented to be from 11% to 41% across this vast country with numerous socio-cultural varieties. In general, the International Diabetes Federation (IDF) estimates that one-quarter of the world's adult population has the MetS [6].

SCH, defined as elevated thyroid-stimulating hormone (TSH) with free T4 concentrations at the lower end of the euthyroid range, affects approximately 4-10% of the general population [7,8]. SCH has been shown to be associated with more severe coronary and carotid artery disease [9-12]. The prevalence of SCH in the United States adult population is 4-8.5%, although this figure increases with age, may differ among ethnic groups and less consistent data is available among men [13,14]. Various other epidemiological studies in India mentioned in this study have shown a prevalence rate varying between 9% and 11.4% [15]. The progression to overt hypothyroidism is approximately 2-5% per year. The rate of progression is proportional to baseline TSH concentration and is higher in individuals with antithyroid antibodies [16].

SCH and MetS are two common endocrine disorders encountered in clinical practice. These two dual conditions when occur together aggravate the complications of each other. The possible reason postulated for the association of SCH and MetS could be of genetic, biochemical or of hormonal origin. Impaired P13k/Akt signaling is a hallmark feature of MetS and thyroid hormone clearly promotes beneficiary changes in this signaling cascade [17]. Therefore, there is a need to evaluate all patients of MetS for SCH. Because of limited data available and few studies have been done in this area, we decided to do this study and find the prevalence of SCH in patients of MetS. If this study proves significant prevalence of SCH in MetS patients, it will open a new vista to study the beneficial effect of treating such patients at earlier stages and help in preventing complications arising out of it.

## Materials and methods

This is a prospective cross-sectional study conducted in Department of General Medicine, New Civil Hospital, Surat, Gujarat, India. The study included 80 subjects, who met the inclusion criteria, willing to give consent and visited Department of General Medicine as outpatients and inpatients during a 22-month period. The patients who fulfilled the criteria for MetS by the National Cholesterol Education Program Adult Treatment Panel III (NCEP ATP III), were taken into study [18]. NCEP ATP III criteria included: central obesity - defined as waist circumference with gender-specific values (for men >102 cm and for women >88 cm). Raised triglycerides: >150 mg/dl. Reduced high-density lipoprotein (HDL) cholesterol: <40 mg/dl in men, <50 mg/dl in women. Raised blood pressure: >130/85 mmHg. Raised fasting blood glucose: >110 mg/dl.

Inclusion criteria

For a person to be defined as having the MetS, they must have more than or equal to any three of the above-mentioned criteria. Patients of age more than 18 years, who fulfilled the criteria were taken into study.

Exclusion criteria

Patients having any liver disorder, renal disorder, or congestive cardiac failure. Patients who are pregnant or on oral contraceptive pills. Patients taking any medication for thyroid-related disorders, statins, or other medications that alter thyroid function and lipid levels. Acute patients were also excluded, considering of sick euthyroid syndrome. Patients not willing to give consent and patients aged <18 years old were also excluded from the study.

A detailed history and anthropometric measurements like height, weight, waist circumference were noted in a semi-structured proforma. Blood pressure was recorded over the right arm with the patient lying supine. After eight hours of fasting, blood was drawn for fasting blood glucose, lipid profile, and thyroid profile (free T3, free T4, and serum TSH) in a single sitting. The lipid profile was done in ERBA CHEM-7 (Transasia Bio Medical Ltd, Baddi, HP) machine, which used the cholesterol oxidase-p-aminophenazone (CHOD-PAP) method for serum cholesterol, glycerol-3-phosphate oxidase (GPO) method for triglycerides and direct method for low-density lipoprotein (LDL) cholesterol and high-density lipoprotein (HDL) cholesterol. The thyroid profile (free T3, Free T4, and TSH) was done on Siemens ADVIA Centaur XP (Siemens Healthineers, Erlangen, Germany) by chemiluminescence immunoassay (CLIA) method.

Statistical analysis

The analysis included profiling of patients on different demographic, clinical and laboratory parameters. Quantitative parameters were tested for normality using the Kolmogorov-Smirnov test. Descriptive analysis of quantitative parameters was expressed as means and standard deviation. Categorical data were expressed as absolute number and percentage. Cross tables were generated, and chi-square test was used for testing of association of categorical variables. Independent student t-test was used for testing of the mean value of study parameters between independent groups. P-value<0.05 was considered statistically significant. All analysis were done using SPSS software version 24.0.

## Results

This study was conducted in the Department of General Medicine, in Government Medical College, Surat from December 2018 to September 2020, where 80 patients of MetS were included in the study. The prevalence of SCH in patients of MetS was studied.

In the present study, female patients were - 48 (60.0%) and males were 32 (40.0%). The mean ± SD age of the patients was 46.5±9.5 years (Range: 24-65 years) and median (IQR) age was 45 years (IQR: 40-54). Majority of the patients belongs to the age group (41-50) years i.e., 29 (36.3%). Age of the women ranges from 24 to 64 years with mean ± SD age - 45.8±9.4 years and median (IQR) - 45 (IQR: 40-53) years. Age of the men ranges from minimum of 26 to maximum of 65 with mean ± SD age - 47.6±9.5 years and median (IQR) age - 48 (IQR: 42-54) years (Table 1).

**Table 1:** Population characteristics SD: standard deviation; IQR: interquartile range Values are mentioned in n (%).

Age (years)	Total (n=80)	Male (n=32)	Female (n=48)
<30	4 (5%)	2 (6.3%)	2 (4.2%)
31-40	20 (25%)	5 (15.6%)	15 (31.3%)
41-50	29 (36.3%)	13 (40.6%)	16 (33.3%)
51-60	22 (27.5%)	9 (28.1%)	13 (27.1%)
>60	5 (6.3%)	3 (9.4%)	2 (4.2%)
Mean ± SD (Range)	46.5±9.5 (24-65)	47.6±9.5 (26-65)	45.8±9.4 (24-64)
Median (IQR)	45 (40-54)	48 (42-54)	45 (40-53)

According to the NCEP ATP III criteria among patients of MetS, 61 (76.3%) patients found to be having euthyroid, 15 (18.8%) patients had SCH, three (3.8%) patients had overt hypothyroidism, and one (1.3%) patient had subclinical hyperthyroidism. There were no overt hyperthyroid patients in our study. There was no significant difference observed of thyroid status distribution with respect to gender in present study (Table 2).

**Table 2:** Gender-wise thyroid status of the study population Values are mentioned in n (%).

Thyroid status	Male (n=32)	Female (n=48)	Total number of patients (n=80)
Euthyroid	25 (78.1%)	36 (75%)	61 (76.2%)
Overt hypothyroidism	1 (3.1%)	2 (4.2%)	3 (3.8%)
Subclinical hypothyroidism	6 (18.8%)	9 (18.8%)	15 (18.8%)
Subclinical hyperthyroidism	0 (0%)	1 (2.1%)	1 (1.2%)

In the age group >60 years, there were five patients, and all were euthyroid. Among 22 patients from age group 51-60 years, six (27.3%) patients had SCH. In the age group 41-50 years, there were 29 patients, among them six (20.7%) patients had SCH. Among 20 patients of the age group 31-40 years, two (10%) patients were found to have SCH. In the age < 30 years, there were four patients, among them three (75%) were euthyroid and one (25%) patient had SCH (Table 3).

**Table 3:** Age-wise thyroid status of the study population Values are mentioned in n (%).

Thyroid status	<30 years (n=4)	31-40 years (n=20)	41-50 years (n=29)	51-60 years (n=22)	>60 years (n=5)	Total (n=80)
Euthyroid	3 (75%)	17 (85%)	20 (69%)	16 (72.7%)	5 (100%)	61 (76.3%)
Overt hypothyroidism	0 (0%)	1 (5%)	2 (6.9%)	0 (0%)	0 (0%)	3 (3.8%)
Subclinical hyperthyroidism	0 (0%)	0 (0%)	1 (3.4%)	0 (0%)	0 (0%)	1 (1.3%)
Subclinical hypothyroidism	1 (25%)	2 (10%)	6 (20.7%)	6 (27.3%)	0 (0%)	15 (18.8%)

Based on the MetS criteria, 38 (47.5%) patients who fulfilled three of the five parameters, 26 (68.4%) had euthyroid status and 12 (31.6%) had SCH; of the 27 (33.8%) patients who fulfilled four parameters, 23 (85.2%) patients had euthyroid and 2 (7.4%) patients had SCH status; of the 15 patients who met all five parameters, 12 (80%) patients had euthyroid status and 1(6.7%) patient had SCH. It is shown in Table 4.

**Table 4:** Metabolic syndrome parameters wise thyroid dysfunction Values are mentioned in n (%).

Thyroid status	Number of parameters of metabolic syndrome			
	3 (n=38)	4 (n=27)	5 (n=15)	Total (n=80)
Euthyroid	26 (68.4%)	23 (85.2%)	12 (80%)	61 (76.3%)
Overt hypothyroidism	0 (0%)	1 (3.7%)	2 (13.3%)	3 (3.8%)
Subclinical hypothyroidism	12 (31.6%)	2 (7.4%)	1 (6.7%)	15 (18.8%)
Subclinical hyperthyroidism	0 (0%)	1 (3.7%)	0 (0%)	1 (1.3%)

The difference observed in mean age (years), mean BMI (kg/m^2^), and mean waist circumference (cm) between patients who had subclinical hypothyroidism and other types of thyroid dysfunction was not found to be statistically significant with p-value of 0.902, 0.306 and 0.988 respectively. Also, there was no statistically significant difference observed in mean systolic blood pressure (SBP) (mm Hg) and diastolic blood pressure (DBP) (mm Hg) between patients who had SCH and other types of thyroid dysfunction (p-value> 0.05) (Table 5).

**Table 5:** Comparison of the mean value of demographic and anthropometric parameters, SBP, and DBP between thyroid dysfunction groups SD: standard deviation; SE: standard error; CI: confidence interval; BMI: body mass index; WC: waist circumference; SBP: systolic blood pressure; DBP: diastolic blood pressure p-value <0.05 is significant.

	Subclinical hypothyroidism mean±SD (n=15)	Others mean±SD (n=65)	(Mean ± SE) of difference	95% of CI of the difference		t-value	p-value
				Lower	Upper		
Age (Years)	46.8±8.9	46.4±9.7	0.3±2.7	-4.97	5.62	0.123	0.902
Height (cm)	157.8±7.2	158.7±8.8	-0.9±2.4	-5.63	3.84	-0.374	0.709
Weight (kg)	80.7±10.8	79.2±10.5	1.5±3	-4.40	7.37	0.502	0.617
BMI (kg/m^2^)	32.3±3	31.4±3.2	0.9±0.8	-0.85	2.67	1.031	0.306
WC (cm)	101.1±8.8	101.1±7.1	0.02±2.1	-4.17	4.11	-0.015	0.988
SBP (mmHg)	132±7.8	132.7±6.7	-0.7±1.9	-4.55	3.18	-0.354	0.724
DBP (mmHg)	86.3±4.8	86.9±4.5	-0.7±1.3	-3.22	1.84	-0.541	0.590

As shown in Table 6, HDL (mg/dl) and TSH(mIU/l) were significantly higher in the SCH group as compared to another type of hypothyroidism group (p-value < 0.05).

**Table 6:** Comparison of mean value of laboratory parameters between thyroid dysfunction groups SD: standard deviation; SE: standard error; CI: confidence interval; FBS: fasting blood sugar; HDL: high-density lipoprotein; TSH: thyroid-stimulating hormone p-value <0.05 is significant.

	Subclinical hypothyroidism mean±SD (n=15)	Others mean±SD (n=65)	(Mean ± SE) of difference	95% of CI of the difference		t-value	p-value
				Lower	Upper		
FBS (mg/dl)	146.1±19.1	139.6±22	6.5±6	-5.41	18.51	1.090	0.279
Total cholesterol (mg/dl)	233.5±30.6	235.2±32.2	-1.7±8.9	-19.48	16.02	-0.195	0.846
HDL (mg/dl)	54.3±7.2	49.2±8.8	5.1±2.4	0.39	9.89	2.156	0.034
Triglycerides (mg/dl)	158.1±31.5	168.5±31	-10.4±8.7	-27.68	6.93	-1.194	0.236
Free T3 (pg/ml)	3.13±0.42	3.13±0.51	0±0.14	-0.27	0.28	0.017	0.987
Free T4 (ng/dl)	1.13±0.11	1.16±0.26	-0.02±0.07	-0.16	0.11	-0.359	0.720
TSH (mIU/l)	6.7±2	2.7±5.9	3.9±1.5	0.97	6.91	2.642	0.010

## Discussion

MetS is characterized by central obesity, hypertriglyceridemia, low HDL cholesterol, hypertension, and abnormal fasting blood glucose levels. MetS can be associated with endocrine and non-endocrine disorders and has widespread consequences. Alterations in thyroid functions, though well known, are not recognized clinically and there is inconsistency in thyroid functions in MetS [19]. MetS and risk factors leading to it increase the risk of developing CVD and type 2 diabetes mellitus. SCH, defined as elevated TSH with free T4 concentrations at the lower end of the euthyroid range, has been shown to be associated with more severe coronary and carotid artery disease [9-12]. SCH and MetS when occur together, aggravate the complications of each other. Thyroid hormone levels can affect HDL, triglycerides, and total cholesterol levels.

In this study among 38 (47.5%) patients who fulfilled three parameters of MetS, 12 (31.6%) had SCH. Of 27 (33.8%) patients who fulfilled four parameters, two (7.4%) patients had SCH. Among 15 (18.8%) who fulfilled all five criteria for MetS, one (6.7%) patient had SCH. So, among 80 patients, found to have MetS as per NCEP ATP III criteria, 15 (18.8%) patients were found to be having SCH. In total hypothyroidism is 22.6% prevalent in MetS patients (overt hypothyroidism 3.8%, SCH 18.8%). No patients in our study had overt hyperthyroidism. Of 22 patients who were in the age group 51-60 years, six (27.3%) patients had SCH. There were 29 patients in the age group 41-50 years, among them six (20.73%) patients had SCH. From 20 patients in the 31-40 years age group, two (10%) patients had SCH. In the age <30 years, there were four patients, among them one (25%) patient was having SCH. None of the five patients from the >60 years age group had SCH. HDL (mg/dl) and TSH (mIU/l) were significantly higher in the subclinical hypothyroidism group as compared to another type of hypothyroidism group (p-value < 0.05).

As observed in our study, there is a statistically significant prevalence of SCH (18.8%) in MetS patients. In this study, one-fifth of MetS patients, or every fifth of patients with MetS have SCH. Impaired P13K/AKT signaling is a hallmark feature of MetS, and thyroid hormone clearly promotes beneficial effects in this signaling cascade [17]. There is a justification for not only proactively looking for SCH in MetS but also treating it, providing new vista and concept. It is important to note that, 2-5% of patients having SCH transform to overt hypothyroidism in a year [16].

Even a small weight loss (3% or more) results in improvement of MetS components. The most likely explanation for that is a higher turnover rate of intra-abdominal fat deposits [20,21]. In fact, weight loss is an unmet need of MetS patients. In such patients, SCH is an important factor that has been missed and when treated, helps to reduce the weight. Continuous treatment with a disciplined diet, physical activity, emotional control, and adherence to therapy results in up to 10% reduction in weight and reversal of MetS. Treatment with thyroxine in MetS with SCH reverts two important components of MetS i.e. central obesity and triglycerides level [22].

Various chemical studies, Saito et al. [23], Kotsis et al. [24], and a meta-analysis by Cai et al. [25], have shown a link between hypothyroidism and a significant increase in SBP and DBP. The mechanisms responsible are peripheral vascular resistance, renal dysfunction, volume changes, and endothelial dysfunction. In MetS patients where SCH is existing and treated, helps to reduce one more component of hypertension in MetS patients. SCH causes a decrease in HDL and a small increase in LDL. Treating SCH in MetS patients helps to ameliorate HDL components and potential adverse cardiac events [26].

Looking proactively for SCH in MetS and treating it would not only prevent overt hypothyroidism what is quaternary prevention but open a new vista for treating MetS patients with possible earliest diagnosis of SCH by community screening and correcting it to reverse MetS. Where it is not possible, at least treating SCH effectively as discussed above, prevents complications of MetS. More such studies in co-relation with metabolic effects may result in a successful and standard universally acceptable algorithmic protocol for MetS treatment.

Limitations

We acknowledge that the study’s uncontrolled design and the sample size of 80 participants may limit the statistical power and introduce selection bias due to the single-center setting. These limitations should be considered when interpreting the findings, which are intended as preliminary insights rather than definitive conclusions. The decision to use this design was based on the exploratory nature of the study, aiming to identify trends and generate hypotheses for future, more comprehensive research. While the single-center approach allowed for consistency in data collection, we recognize that larger, controlled studies with diverse populations are necessary to validate and generalize these results. Despite these constraints, we believe the study provides valuable initial data that can guide future investigations.

## Conclusions

To conclude, the prevalence of thyroid dysfunction in patients with MetS was high, indicating a possible interplay between thyroid status and MetS. SCH is the most common thyroid dysfunction in patients with MetS, especially females who are at increased risk. Although thyroid hormone significantly affects each component of MetS, there was no relationship between thyroid and all components of MetS. The data generated from the present study will aid in establishing a correlation between thyroid dysfunction and MetS in Indian patients. This data will have prognostic importance for practitioners in their routine clinical practice to develop strategies for better management of their patients of thyroid dysfunction with associated MetS. This early diagnosis of thyroid dysfunction in MetS would help in modifying the disease course by early interventions with appropriate lifestyle modification regimens, as applicable. However, future large sample-sized prospective studies are warranted which could evaluate the impact of thyroid dysfunction management in terms of reduction in MetS and its related components.
